# Association between perioperative flurbiprofen administration and acute kidney injury (AKI) in spine surgery: a retrospective cohort study

**DOI:** 10.1186/s13741-024-00419-2

**Published:** 2024-06-18

**Authors:** Yongrong Liu, Bo Li, Lihua Hang, Li Zhang

**Affiliations:** 1 Department of Operation Center, The Third Xiangya Hospital of Central South University, Changsha, 410013 China; 2 Department of Anesthesiology, The First People’s Hospital of Kunshan Affiliated With Jiangsu University, Kunshan, 215300 China

**Keywords:** Flurbiprofen, NSAID, AKI, Spine surgery

## Abstract

**Background:**

The association between nonsteroidal anti-inflammatory drugs (NSAIDs) and postoperative acute kidney injury (AKI) remains controversial, with limited studies specifically examining flurbiprofen. Therefore, this research aimed to investigate the association between intraoperative flurbiprofen administration and postoperative AKI.

**Methods:**

We retrospectively identified a cohort of patients at the Third Xiangya Hospital of Central South University. A total of 3882 adult patients undergoing spinal surgery between January 1, 2012, and July 31, 2018, were included and classified into two groups: those receiving flurbiprofen (50 or 100 mg once, 5 min after anesthesia start) and those not receiving flurbiprofen. The primary endpoint was the incidence of AKI.

**Result:**

The flurbiprofen group (4.4%) had a lower incidence of AKI compared to the non-flurbiprofen group (6.5%, *P* < 0.001). After adjusting for potential confounding variables, the multivariable regression analysis showed that the flurbiprofen group had a 49% reduced risk of postoperative AKI (OR 0.51; 95% CI 0.31 to 0.82) compared to the non-flurbiprofen group. Subgroup analysis indicated that flurbiprofen injection was associated with a reduced incidence of postoperative AKI in patients without diabetes (OR 0.61; 95% CI 0.19 to 0.74), surgical times of 2–5 h (OR 0.54; 95% CI 0.23 to 0.75), and preoperative anemia (OR 0.57; 95% CI 0.21 to 0.74).

**Conclusion:**

The study concluded that perioperative flurbiprofen treatment was associated with a lower risk of postoperative AKI in adult patients undergoing spinal surgery.

## Introduction

Nonsteroidal anti-inflammatory drugs (NSAIDs) are extensively utilized for postoperative analgesia and are included in the World Health Organization's pain ladder (Lubis et al. 2021; Mittal et al. 2022). Despite their benefits, NSAIDs are associated with an increased risk of bleeding, cardiovascular events, and acute kidney injury (AKI) (O'Connor et al. 2016; Non-steroidal anti-inflammatory drugs and risk of heart failure in four European countries: nested case–control study. 2016; Hakkarainen et al. 2015).

Flurbiprofen, a propionic acid derivative of NSAIDs, inhibits cyclooxygenase (Cox-1 and -2), reducing prostaglandin synthesis which plays a crucial role in pain and inflammatory pathways (Wang et al. 2017). Consequently, flurbiprofen possesses analgesic, antipyretic, and anti-inflammatory properties (Wang et al. 2017).

Acute kidney injury (AKI) is a significant concern among surgical patients, affecting 6–8% (Biteker et al. 2014; Mizota et al. 2017) of this population, and is linked with increased mortality and the risk of chronic kidney disease (Heung et al. 2016). The primary diagnostic criterion for AKI is a reduction in the indirectly measured glomerular filtration rate (GFR) (Huo et al. 2022). Previous research has underscored the prognostic significance of AKI diagnosis, particularly highlighting its predictive value for readmission following lumbar surgery for lumbar spinal stenosis (Ilyas et al. 2019). Additionally, recent studies have suggested that low-dose flurbiprofen administration may reduce postoperative AKI incidence (Wang et al. 2020). Despite these insights, data on the impact of flurbiprofen remains sparse. To address these knowledge gaps, our study uniquely focuses on patients undergoing spinal surgery, a population characterized by distinct features and risk factors. Furthermore, we utilize propensity score matching (PSM) analysis to mitigate the influence of potential confounding variables between the treatment and control groups. By doing so, our aim is to provide compelling evidence elucidating the relationship between perioperative flurbiprofen treatment and postoperative AKI in spinal surgery patients.

## Methods

### Design and selection criteria

This study received approval from the ethics committee of the Third Xiangya Hospital at Central South University (2018-S376). The requirement for informed consent was waived, and all data were sourced from electronic medical records. Adult patients undergoing spine surgery from January 1, 2012, to July 31, 2018, were included. Exclusion criteria encompassed patients with ASA grade IV–V, those receiving regional anesthesia from a surgeon, administration of other NSAIDs, absence of serum creatinine, and preoperative CKD with an eGFR below 90 mL/min/1.73 m^2^. Flurbiprofen was administered in a single dose of 50 or 100 mg, 5 min following anesthesia initiation. Patients receiving over 100 mg of flurbiprofen were excluded.

### Data collection

Information collected includes (1) demographic data (age and gender); (2) baseline history (preoperative complications, medications); (3) laboratory results (serum creatinine, eGFR calculated using the CKD Epidemiology Collaboration formula); (4) intraoperative details (emergency status, surgical time, type of anesthesia, ASA grade, fluid management, erythrocyte transfusion, blood loss); and (5) postoperative outcomes (incidence and severity of AKI). Multiple imputation will be employed to address missing data when less than 5% of a variable is missing.

### Definitions

The primary endpoint was the occurrence of AKI within 7 days post-spine surgery, assessed and classified according to the Kidney Disease Improving Global Outcomes (KDIGO) standards (Lameire et al. 2021). AKI was defined as an increase in serum creatinine of more than 1.5 times from baseline within seven postoperative days or more than 0.3 mg/dL increase within 48 h. Postoperative AKI was classified into four stages according to the KDIGO: stage 0, no AKI; stage 1, AKI grade 1; stage 2, AKI grade 2; and stage 3, AKI grade 3.

Flurbiprofen injection was defined as taking 50 or 100 mg in 5 min after anesthesia started. The lowest preoperative creatinine value post-admission was used to calculate eGFR and the baseline CKD stage was measured using the Chronic Kidney Disease Epidemiology Collaboration algorithm (Levey et al. 2009). Out fluid amount refers to the total volume of urine and blood loss within a 24-h period.

### Statistical analysis

Eligible participants in the two groups had different baseline characteristics (Table 1). Propensity-score matching, conducted in a 1:2 ratio, identified a cohort of patients with similar baseline characteristics. The matching utilized the greedy-matching algorithm and included age, sex, and eGFR as covariates. The caliper width was equal to 0.2 of the standard deviation of the logit of the propensity score.

**Table 1:** Baseline characteristics before and after propensity score matching

Characteristic	Before matching			After matching		
	Control group (*n* = 3093)	Flurbiprofen group (*n* = 789)	*P*	Control group (*n* = 1324)	Flurbiprofen group (*n* = 662)	*P*
Age (year)	53.37 ± 14.62	50.95 ± 13.81	** < 0.001**	48.57 ± 10.45	48.48 ± 10.46	0.867
Male, *n* (%)	1683 (54.4)	411 (52.10)	0.236	681 (51.4)	337 (50.8)	0.819
Smoking, *n* (%)	448 (14.5)	92 (11.6)	**0.032**	52 (3.9)	76 (11.8)	< **0.001**
Alcohol consumption, *n* (%)	285 (9.2)	59 (7.5)	0.142	28 (2.1)	48 (7.3)	** < 0.001**
Hypertension, *n* (%)	637 (20.6)	118 (14.9)	** < 0.001**	175 (13.2)	85 (12.9)	0.828
Diabetes, *n* (%)	204 (6.9)	50 (6.4)	0.651	79 (6)	42 (6.3)	0.802
ACEI, *n* (%)	158 (5.1)	27 (3.4)	0.052	41 (3.1)	21 (3.1)	0.973
CCB, *n* (%)	780 (25.2)	137 (17.4)	** < 0.001**	250 (18.9)	99 (14.9)	**0.029**
Diuretics, *n* (%)	68 (2.2)	3 (0.4)	** < 0.001**	19 (1.4)	2 (0.3)	**0.019**
ASA grade, *n* (%)			0.764			0.273
I–II	2626 (84.9)	673 (85.3)		1110 (83.8)	568 (85.7)	
III	467 (15.1)	116 (14.7)		214 (16.2)	947 (14.3)	
Serum creatinine, (μmoI/L)	64 (52,78)	63 (52,74)	0.029	64 (52,79)	63 (53,77)	0.795
eGFR, ml/min/1.73 m^2^	122.7 (85.4,189.7)	116.8 (88.4,191.8)	**0.006**	127 (90.5,185)	129 (92.8,182)	0.906
In fluid amount (100 mL/24 h)	26.0 (16.0, 38.0)	26.0 (16.0, 37.0)	0.723	22.0 (15.0, 34.50)	26.0 (16.0, 37.0)	** < 0.001**
Out fluid amount (100 mL/24 h)	19.0 (4.0, 15.50)	8.50 (4.0, 14.0)	0.65	7.0 (3.0, 13.0)	8.50 (4.0, 14.0)	< 0.001
Surgical time (h)	2.81 ± 1.72	3.04 ± 1.74	** < 0.001**	2.53 ± 1.58	3.02 ± 1.77	** < 0.001**
Preoperative hemoglobin, (g/L)	97.0 (30.4, 139.0)	103.0 (30.5, 152.0)	0.057	98.0 (30.4, 142.0)	104.0 (30.5, 152.0)	0.118
Emergency, *n* (%)	436 (14.1)	68 (8.6)	** < 0.001**	185 (14)	50 (7.6)	** < 0.001**
Intraoperative erythrocyte transfusion, mL (%)			**0.008**			0.135
< 100	2202 (71.2)	608 (77)		1000 (75.5)	520 (78.6)	
100–600	331 (10.7)	69 (8.7)		123 (9.3)	50 (7.6)	
601–1000	269 (8.7)	60 (7.6)		85 (6.4)	48 (7.3)	
> 1000	291 (9.4)	59 (6.7)		113 (8.8)	42 (6.4)	
Intraoperative hemorrhage, mL (%)			** < 0.001**			** < 0.001**
< 100	882 (28.5)	169 (21.4)		459 (34.7)	146 (22)	
100–600	1605 (51.9)	454 (57.5)		649 (49)	381 (57.8)	
601–1000	257 (8.3)	85 (10.8)		94 (7.1)	70 (10.5)	
> 1000	350 (11.3)	80 (10.2)		122 (9.2)	64 (9.7)	
AKI, *n* (%)	201 (6.5)	35 (4.4)	**0.032**	136 (10.3)	30 (4.5)	** < 0.001**
AKI. stages, *n* (%)			0.183			** < 0.001**
0	2895 (93.6)	754 (95.6)		1188 (89.7)	632 (95.5)	
1	102 (3.3)	17 (2.1)		71 (5.4)	15 (2.2)	
2	68 (2.2)	13 (1.7)		48 (3.6)	11 (1.6)	
3	28 (0.9)	5 (0.6)		19 (1.4)	5 (0.7)	

The stages of AKI (stage 0, no AKI; stage 1, AKI grade 1; stage 2, AKI grade 2; and stage 3, AKI grade 3)

*AKI* acute kidney injury, *ARB* angiotensin receptor blockers, *ASA* American Society of Anesthesiologists, *BMI* body mass index, *CCB* calcium-channel blockers, *eGFR* estimated glomerular filtration rate. Data are presented as mean ± SD, median (IQR), or numbers (percentages)

The numbers and percentages were used for categorical variables to summarize patients’ characteristics. The chi-square test or Fisher exact test was used to compare the proportional differences for the summarized categorical data. Continuous data was presented as means ± standard deviations with Kruskall-Wallis tests for omnibus comparisons or *t* tests for normal data.

To quantify the associations between the administration of flurbiprofen and AKI while adjusting for some potential confounders determined by our team’s clinical competence, confounding variables were first chosen based on *p* values of 0.05 in the univariate logistic regression model. To assess the association between the use of flurbiprofen and AKI stages, linear regression was further performed. The association between flurbiprofen administration and AKI was determined by the subgroup analysis between the two groups. The odds ratios (OR) and the corresponding 95% confidence intervals (CI) were used to express effect estimates. *P* less than 0.05 was considered to be the two-sided statistical significance criterion. All statistical analyses were conducted using the R statistical software package and Empowerstats (http://www.empowerstats.com).

## Results

### Study population

**Fig. 1:**
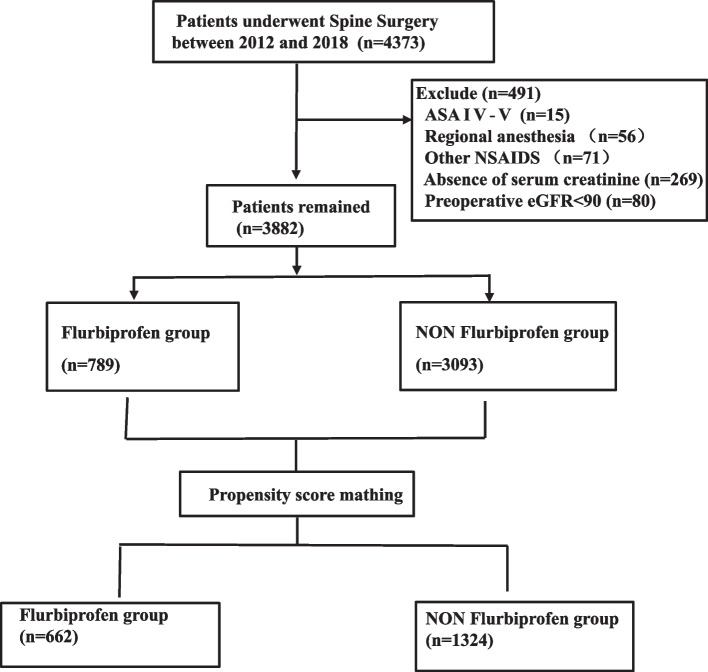
Flowchart of patient selection. Study cohort. Illustration of exclusion and inclusion criteria as utilized to select the final cohort of 1986 patients

A total of 3,882 patients who underwent spine surgery were included in the study, 789 (20.3%) received flurbiprofen and 3093 (79.7%) did not (Fig. 1, Table 1). Baseline variables differed between the two groups prior to propensity-score matching (Table 1). Using propensity-score matching in a 1:2 ratio, 662 individuals receiving flurbiprofen were matched with 1324 patients who did not receive flurbiprofen.

Information on preoperative renal function tests, anesthesia, and surgery for both groups, both before and after propensity score matching, is presented in Table 1. The table showed that some disparities still existed between the two groups after propensity score matching. More participants receiving the flurbiprofen group smoked (11.8% VS 3.9%) and consumed alcohol (7.3% VS 2.1%). Additionally, the flurbiprofen group received more fluids, and fewer diuretics, and were less likely to undergo emergency surgery. However, the flurbiprofen group experienced longer surgery times and more frequent moderate intraoperative blood loss compared to the non-flurbiprofen group. Conversely, non-flurbiprofen group consumed more calcium-channel blockers (CCB) (18.9% VS 14.9%) and diuretics (1.4% VS 0.3%) medicines than flurbiprofen group. The 7-day AKI rate was 4.4% (35/789) in the flurbiprofen group, while 6.5% (201/3093) in the non-flurbiprofen group before matching.

### Univariable analysis

Table 2 indicated that independent associations with postoperative AKI included flurbiprofen use (OR 0.49; 95% CI 0.32 to 0.77, *P* = 0.001), eGFR (OR 1.01; 95% CI 1.01 to 1.02, *P* < 0.001), smoking (OR 2.60; 95% CI 1.53 to 4.44, *P* < 0.001), alcohol consumption (OR 2.60; 95% CI 1.34 to 5.06, *P* = 0.0049), CCB use ( OR 1.66; 95% CI 1.09 to 2.52, *P* = 0.018), non-general anesthesia (OR 0.45; 95% CI 0.23 to 0.87, *P* = 0.016), emergency (OR 1.94; 95% CI 1.22 to 3.07, *P* = 0.005) and intraoperative erythrocyte transfusion > 1000 ml (OR 2.49; 95% CI 1.48 to 4.19, *P* < 0.001). Other variables such as age, sex, hypertension, diabetes, preoperative hemoglobin, ACEI use, diuretic use, surgical duration, and intraoperative hemorrhage showed no association with AKI.

**Table 2:** Univariable analysis for AKI incidence after PSM

Exposure	Statistics	AKI	AKI. Stages
		OR (95% CI) *P*	beta (95% CI) *P*
Flurbiprofen	0.34 ± 0.47	0.49 (0.32,0.77) **0.001**	− 0.09 (− 0.14, − 0.04) **0.001**
Age (year)	48.54 ± 10.45	1.01 (0.99, 1.03) 0.227	0.00 (− 0.00, 0.00) 0.150
Male, *n* (%)	1016 (51.18)	1.34 (0.93, 1.93) 0.118	0.07 (0.03, 0.11) **0.001**
eGFR	136.2 (97.0,187.1)	1.01 (1.01,1.02) < **0.001**	0.01(0.01,0.02) < **0.001**
Smoking, *n* (%)	129 (6.54%)	2.60 (1.53, 4.44) < **0.001**	0.31 (0.22, 0.39) < **0.001**
Alcohol consumption, *n* (%)	76 (3.88)	2.60 (1.34, 5.06)** 0.0049**	0.36 (0.25, 0.47) < **0.001**
Hypertension, *n* (%)	260 (13.09)	1.04 (0.61, 1.76) 0.888	− 0.02 (− 0.08, 0.05) 0.561
Diabetes, *n* (%)	120 (6.09)	0.62 (0.25, 1.55) 0.307	0.04 (− 0.05, 0.13) 0.388
ACEI, *n* (%)	61 (3.12%)	0.74 (0.23, 2.41) 0.623	− 0.04 (− 0.17, 0.08) 0.527
CCB, *n* (%)	349 (17.56%)	1.66 (1.09, 2.52) **0.018**	0.15 (0.09, 0.20) < **0.001**
Diuretics, *n* (%)	21 (1.06%)	1.56 (0.36, 6.79) 0.55	0.01 (− 0.21, 0.22) 0.948
Anesthesia method, *n* (%)			
General	1676 (84.40)	1	0
Non-general	310 (15.60)	0.45 (0.23, 0.87)** 0.016**	− 0.10 (− 0.16, − 0.04) **0.001**
Emergency, *n* (%)	236 (11.88)	1.94 (1.22, 3.07) **0.005**	0.09 (0.02, 0.15) **0.012**
Intraoperative erythrocyte transfusion, mL (%)			
< 100	1521 (76.55)	1	0
100–600	172 (8.71)	1.18 (0.61, 2.25) 0.624	0.08 (0.00, 0.16) 0.046
601–1000	134 (6.74)	1.70 (0.90, 3.21) 0.098	0.05 (− 0.04, 0.14) 0.256
> 1000	159 (8.00)	2.49 (1.48, 4.19) < **0.001**	0.24 (0.16, 0.32) < **0.001**
Intraoperative hemorrhage, mL (%)			
< 100	605 (30.45)	1	0
100–600	1032 (51.99)	0.78 (0.51, 1.19) 0.246	− 0.05 (− 0.10, 0.00) 0.066
601–1000	163 (8.20)	1.22 (0.64, 2.35) 0.542	0.01 (− 0.08, 0.09) 0.864
> 1000	186 (9.36)	1.61 (0.91, 2.85) 0.104	0.08 (− 0.00, 0.16) 0.056
Surgical time (h)	2.70 ± 1.67	1.06 (0.96, 1.17) 0.259	0.00 (− 0.01, 0.02) 0.594
Preoperative anemia, *n* (%)			
0	835 (42.02)	1	0
1	1151 (57.98)	1.28 (0.88, 1.86) 0.195	0.04 (− 0.00, 0.09) 0.060

*AKI* acute kidney injury, *ARB* angiotensin receptor blockers, *ASA* American Society of Anesthesiologists, *BMI* body mass index, *CCB* calcium-channel blockers, *eGFR* estimated glomerular filtration rate are all terms that are used in the medical field. Data are presented as mean ± SD or numbers (percentages)

### Multivariable regression analysis

The results of the multivariable regression analysis are presented in Table 3. Flurbiprofen was independently associated with postoperative AKI (OR 0.51; 95% CI 0.31 to 0.82; *P* = 0.006) and various stages of postoperative AKI (beta 0.08; 95% CI 0.12 to 0.03; *P* < 0.001).

**Table 3:** Association between flurbiprofen use and AKI in patients with spine surgery

	**Non-adjusted**	**Adjust I**	**Adjust II**
	OR/beta (95%CI), *P*	OR/beta (95%CI), *P*	OR/beta (95%CI), *P*
AKI	0.49 (0.32, 0.77) 0.001	0.49 (0.33, 0.79) 0.004	0.51 (0.31, 0.82) 0.006
AKI Stages	− 0.09 (− 0.14, − 0.04) < 0.001	− 0.09(− 0.13, − 0.04) < 0.001	− 0.08 (− 0.12, − 0.03) < 0.001

*Adjust I* age, gender, smoking, alcohol consumption, preoperative anemia, hypertension, diabetes mellitus, ACEI, CCB, and diuretics were all taken into account

*Adjust II* adjust I plus eGFR, emergency, amount of fluid infusion and out, intraoperative erythrocyte transfusion, and amount of blood loss

### Subgroup analysis

Table 4 presents the association between flurbiprofen use and reduced postoperative AKI incidence in specific patient groups. A single dose of flurbiprofen (50–100 mg) was associated with a lower risk of postoperative AKI in non-diabetic patients (OR 0.61; 95% CI 0.19 to 0.74), those with surgical durations of 2–5 h (OR 0.54; 95% CI 0.23 to 0.75), and those with preoperative anemia (OR 0.57; 95% CI 0.21 to 0.74) as shown in Table 4.

**Table 4:** Subgroup analysis of the association between flurbiprofen and postoperative AKI

Variable	Non-adjusted		Adjust I		Adjust II	
	OR (95%CI)	*P*	OR (95% CI)	*P*	OR (95% CI)	*P*
Non- diabetes	0.55 (0.23 ~ 0.55)	< 0.01	0.61 (0.21 ~ 0.73)	< 0.01	0.61 (0.19 ~ 0.74)	< 0.01
Surgical time (2–5 h)	0.54 (0.23 ~ 0.73)	< 0.01	0.53 (0.23 ~ 0.73)	< 0.01	0.54 (0.23 ~ 0.75)	< 0.01
Preoperative anemia	0.50 (0.24 ~ 0.68)	< 0.01	0.51 (0.21 ~ 0.73)	< 0.01	0.57 (0.21 ~ 0.74)	< 0.01

*Adjusted I* for age, sex, smoking, alcohol consumption, hypertension, diabetes mellitus, CCB, Emergency and Anesthesia

*Adjust II* Adjust I plus amount of fluid infusion and out, and intraoperative erythrocyte transfusion

*AKI* acute kidney injury, *CCB* calcium-channel blockers, *eGFR* estimated glomerular filtration rate

## Discussion

In this study, we explore the effects of flurbiprofen on postoperative acute kidney injury (AKI). While previous research has indicated an association between nonsteroidal anti-inflammatory drugs (NSAIDs) and an increased risk of AKI, our findings demonstrate that the perioperative use of low-dose Flurbiprofen in spinal surgeries is associated with a reduced risk of AKI.

Observational studies present conflicting results concerning the relationship between NSAID usage and AKI (Chiu et al. 2015; Zhan et al. 2020; Drugs and (NSAID) administration and acute kidney injury (AKI) in major gastrointestinal surgery: a prospective, multicenter, propensity matched cohort study. 2020; Aboul-Hassan et al. 2020). For instance, a study involving over 12,000 patients with rheumatoid arthritis (RA) found an association between NSAID use and increased risk of chronic kidney disease (CKD) (Chiu et al. 2015). However, recent findings indicate that short-term use of NSAIDs early in the postoperative period does not correlate with increased AKI risk within the first seven days postoperatively (Drugs and (NSAID) administration and acute kidney injury (AKI) in major gastrointestinal surgery: a prospective, multicenter, propensity matched cohort study. 2020). Additionally, the use of ibuprofen has been linked to a reduced risk of AKI, consistent with the results of our current investigation (Drugs and (NSAID) administration and acute kidney injury (AKI) in major gastrointestinal surgery: a prospective, multicenter, propensity matched cohort study. 2020). In addition, according to a recent study, continuation of aspirin administration after coronary artery bypass grafting was linked to a lower risk of acute kidney injury (AKI) than the interruption of aspirin 24 to 48 h before surgery (Aboul-Hassan et al. 2020). In a retrospective observational study with 913 patients who underwent laparoscopic or robot-assisted laparoscopic renal resection, no correlation was observed between NSAID use for PCA and the incidence of postoperative renal impairment (Han et al. 2020).

While it remains unclear how Flurbiprofen reduces the risk of postoperative AKI, inflammation may play a contributory role. Epidemiological studies have shown that chronic inflammation significantly contributes to the progression of AKI to CKD (Sato and Yanagita 2018). Furthermore, research indicates that inflammation predicts postoperative AKI in non-cardiac surgeries and that anti-inflammatory treatments could improve AKI prognosis (Murashima et al. 2019). Flurbiprofen reduces both local and systemic inflammatory cytokines during the postoperative period (Zhao et al. 2021; Esme et al. 2011).

The use of NSAIDs as adjunct analgesics in the postoperative setting may offer significant benefits (Wattchow et al. 2009; Coloma et al. 2000). Although opioids are effective in pain management, they are associated with risks such as long-term dependence, respiratory depression, upregulation of pro-inflammatory signals, and delayed recovery of bowel function (postoperative ileus). NSAIDs have opioid-sparing effects, which could mitigate some of the adverse effects associated with opioids (Martinez et al. 2017). Additionally, the anti-inflammatory properties of NSAIDs may be beneficial throughout the recovery period (Fang et al. 2013) as surgical stimulation increases systemic inflammatory responses, potentially linked with increased short-term and long-term adverse events (Neal et al. 2014; Kalff et al. 2003).

The study has certain limitations that merit discussion. Notably, it employs a retrospective design which inherently suffers from recall bias due to its reliance on past events. Furthermore, the accuracy and completeness of the medical records system serving as the data source may lend an element of uncertainty to the results. Crucial variables such as BMI, intraoperative hypotension, and vasoactive drug usage were unattainable due to the retrospective design, potentially impacting our analysis. Despite efforts to address these factors in data interpretation, the observational nature introduces potential confounding variables. A notable limitation in our study arises from the inability to confirm several typical independent preoperative risk factors for postoperative AKI, partially attributable to data loss affecting the comprehensive assessment of certain variables. However, the introduction of propensity score matching, while capable of reducing bias and confounding variables, also brings forth new sources of bias. It is crucial to underscore that our study establishes an association, not a causal relationship, between AKI incidence and flurbiprofen use. While other studies have looked at low-dose (50–100 mg), medium-dose (100–250 mg), and high-dose (≥ 250 mg) flurbiprofen treatment, our analysis focused solely on the low-dose group. This limitation may affect the comprehensive assessment of treatment effects across different dosage levels. Future research could broaden the analysis to include additional dosage ranges and explore dose-dependent effects further.

## Conclusions

In our study, flurbiprofen preparations are associated with the incidence of postoperative AKI. Compared to non-flurbiprofen group, the use of low-dose flurbiprofen was linked to a decreased risk of AKI. However, given that it is a preliminary observational result, randomized controlled trials should be conducted to further confirm our findings. Unlike some certain pain management techniques that require specialized equipment (such as postoperative monitoring pumps or patient-controlled analgesia in high dependency units), flurbiprofen is affordable and widely accessible, which is especially desirable for usage in settings with limited resources.

## Data Availability

No datasets were generated or analysed during the current study.
